# The trajectory and influencing factors of health-promoting behavior in patients after aortic dissection surgery: a longitudinal study

**DOI:** 10.3389/fpubh.2026.1640998

**Published:** 2026-07-22

**Authors:** Zhengkun He, Haiyan Pei, Qu Chen, Chaolin Fan, Zhonggui Shan, Yizhen Wang, Yan Chen

**Affiliations:** 1 Department of Cardiac Surgery, The First Affiliated Hospital of Xiamen University, Xiamen, Fujian, China; 2 Neurocritical Care Unit, The First Affiliated Hospital of Zhengzhou University, Zhengzhou, Henan, China; 3 Department of Orthopedic Sports Medicine, The First Affiliated Hospital of Xiamen University, Xiamen, Fujian, China

**Keywords:** aortic dissection, GMM, health-promoting behavior, longitudinal study, trajectory

## Abstract

**Background:**

Health-promoting behavior are important in improving health outcome indicators for patients after aortic dissection surgery. However, there has been limited research on the trajectory of health-promoting behavior among Chinese aortic dissection patients.

**Aims:**

To explore the trajectory of health-promoting behavior in patients after aortic dissection surgery and identify influencing factors.

**Methods:**

This prospective longitudinal study included patients who underwent aortic dissection at the cardiovascular surgery of Xiamen Hospital from August to December 2024. Patients with aortic dissection were evaluated at discharge (T0), 1 month (T1), 3 months (T2), and 6 months (T3) after the operation. Using the health-promoting behavior scale evaluated health-promoting behavior. Growth mixture model (GMM) analysis was used to identify heterogeneous health-promoting behavior subgroups in the four observation periods. Univariate and logistic regression analyses were used to identify factors affecting health-promoting behavior classification and independent risk factors.

**Results:**

We identified three health-promoting behavior trajectories among aortic dissection patients: low-stability group (33.80%), medium-volatility group (43.20%) and high-increasing group trajectory groups (23%). Compared to the low-stability group, logistic regression analysis indicates that in the medium-volatility group, age (OR = 0.950, 95%CI: 0.925–0.975, *p* < 0.001) and having a history of hypertension (OR = 1.941, 95%CI: 1.049–3.590, *p* = 0.035) were significant influencing factors. The comparative Logistic regression analysis results between the high-increasing group and the low-stability group showed that age (OR = 0.923, 95%CI: 0.892–0.954, *p* < 0.001), educational level (OR = 2.507, 95%CI: 1.503–4.182, *p* < 0.001) and a history of hypertension (OR = 6.613, 95%CI: 3.056–14.313, *p* < 0.001) were both significant influencing factors.

**Conclusion:**

We identified three health-promoting behavior trajectories, highlighting a significant heterogeneity in health-promoting behavior development. Understanding the characteristics of these developmental trajectories is crucial for realizing the dynamics of different health-promoting behavior subgroups and informing effective interventions.

## Introduction

Aortic dissection (AD) is a disease in which the intima of the aorta tears from part of the media under the impact of local velocity and local pressure blood flow (1). The blood flow passes through the tear cavity, dividing the aortic lumen into a true lumen and a false lumen, with one or more tear openings present (2). Aortic dissection can lead to organ damage, including renal failure and intestinal injury, stroke, aortic valve injury and pericardial tamponade, which may eventually result in disability and/or death (3). The global reported incidence of aortic dissection ranges from 2.5 to 17.6 per 100,000 person-years (4). In recent years, with the continuous development of medical technology, the survival rate of AD patients treated by surgery has been increasing year by year (5). However, there are still high-risk factors for aortic diseases and related cardiovascular events in patients, which seriously reduce the quality of life of patients and bring a large economic burden to individuals and society (6).

Studies have shown that effective health-promoting behavioral interventions for patients with cardiovascular disease can improve cardiac function, enhance quality of life, and reduce the risk of disease recurrence (7–9). Health-promoting behavioral refers to a series of activities taken by individuals to achieve optimal health and realize self-worth (10). The areas of health promoting behaviors include nutrition, physical activity, stress management, health responsibilities, interpersonal relationships, and spiritual growth (11). Health promoting behaviors that have been recommended for patients with AD include dietary control, physical activity, cessation of smoking, medication adherence, and adherence to medical recommendations (including monitoring blood pressure and body weight daily) (12). Therefore, an in-depth understanding of the factors affecting health promotion behaviors in patients with aortic constriction is important for developing targeted interventions to improve their health outcomes.

A qualitative study using the COM-B model identified key factors influencing health-promoting behaviors: psychological abilities (e.g., lack of disease knowledge, limited physical functions, fatigue), physical opportunities (e.g., limited access to information, doctor-patient communication, objective condition limitations), and motivations (e.g., self-efficacy, perceived benefits, personal and family responsibilities) (13). However, there are still deficiencies in the research in this field at present. Firstly, there are relatively few quantitative and longitudinal studies on the health behaviors of AD patients, making it difficult to comprehensively evaluate the long-term effects of health behaviors. Secondly, while most existing health promotion programs are based on the theory of health behavior change, further exploration is still needed regarding specific intervention measures and effect evaluation (14). At the same time, it is not clear what the trajectory characteristics and population heterogeneity of health promotion behaviors in patients with aortic dissection are, as well as the factors related to the trajectory of health promotion behaviors.

Therefore, this study adopted the longitudinal research method to deeply understand the changes in health promotion behaviors of patients with aortic dissection at discharge, 1 months, 3 months, and 6 months after discharge. The GMM model is used to identify the categories of trajectories, and the influencing factors of the changes in patients’ health promotion behaviors are analyzed to provide a basis for implementing personalized behavior management intervention measures.

## Methods

### Design and data collection

The current follow-up study was carried out in Xiamen City, Fujian Province, China. Participants were recruited via a convenience sampling approach from the patient population at the First Affiliated Hospital of Xiamen University. Their participation was determined by their clinical condition and willingness to participate in the study. Eligible participants were identified based on the following criteria: diagnosis of type A or B aortic dissection or in accordance with the diagnostic criteria established by the European Society of Cardiology in 2014 (15); completion of aortic dissection or surgery and subsequent transfer from the intensive care unit to the cardiac and macrovascular surgical unit; age of 18 years or above; and provision of informed consent for participation in the study. Patient exclusion criteria encompassed: postoperative instability (e.g., delirium) that hinders the participant from cooperating in questionnaire completion; a history of mental illness that may affect adherence and health-related behaviors, with specific types of mental illness considered for exclusion including severe depression, schizophrenia, and other conditions that may substantially impact the participant’s ability to follow study protocols and engage in health-related activities; and serious communication issues that prevent the participant from comprehending or responding to the questionnaire.

A uniformly trained researcher elucidated the purpose, methodology, and significance of the study to the respondents. Subsequently, the questionnaire was administered subsequent to obtaining informed consent. In this study, a total of four surveys were carried out. These surveys were, respectively, conducted at the time of patients’ discharge from the hospital (T0), and at 1 month (T1), 3 months (T2), and 6 months (T3) after discharge during outpatient clinic and ward reviews for follow-up. The aim was to collect patients’ general information, disease-related information, and health-promoting behaviors. The researcher pre-retained the contact information of the patients and their families and made prior appointments with the patients for follow-up. The patients completed the questionnaires during the review, which approximately took 15–20 min. Throughout this process, the researcher was on-site to assist participants in comprehending the items, thereby minimizing the probability of data loss or incomplete responses. The term “invalid questionnaires” pertains to those questionnaires that contained missing or illogical answers and were thus unfit for data analysis. The researcher promptly verified the validity of the data, and invalid questionnaires were excluded. At the conclusion of the study, all data were meticulously re-examined for accuracy and completeness. Subsequently, the data were input into a computerized database using a standardized data entry form formulated to minimize errors. Each entry was cross-verified by a second researcher to ensure the reliability and integrity of the data.

### Sample size calculation

The minimum sample size was computed using G*Power 3.1.9.7 software. The parameters incorporated an effect size of 0.1, a significance level (*α*) of 0.05, and a statistical power (1 − *β*) of 0.8. The intra-group correlation coefficient was set at 0 since no pre-survey was carried out, and the non-spherical correlation was set to the default value of 1. Based on these settings, the requisite sample size was ascertained to be 277. In this study, 278 patients completed the 4 follow-up sessions, thereby fulfilling the minimum sample size requirement.

## Measures

### Sociodemographic data

We conducted the survey using a self-administered questionnaire to gather sociodemographic information from students, which included: age, gender, education, occupation, residence, status of employment, marital status, per capita household income, type of medical insurance, type of AD, history of hypertension and history of diabetes.

### Health-promoting behavior scale

This study employed a health-promoting behavior scale for patients with aortic dissection, developed by Tu et al. (16). This scale is a five-point liket scale (1 = never, 2 = seldom, 3 = sometimes, 4 = often, 5 = always), consisting of 25 items and five dimensions: nutrition (six items, score range of 6–30), exercise (six items, score range of 6–30), blood pressure management (five items, score range of 5–25), disease knowledge (five items, score range of 5–25), and behavioral motive (three items, score range of 3–15). The total scores range from 25 to 125, with higher scores indicating better health-promoting behaviors. The scale shows good reliability, with a Cronbach’s *α* coefficient of 0.842.

### Ethics approval and consent to participate

The study was approved by the Ethics Committee of the Affiliated Hospital Ethics Committee of Xiamen University (2022-023). Informed consent was obtained from all participants, and procedures were conducted according to the Declaration of Helsinki. All participants had the right to withdraw at anytime without any adverse effect on the clinical work.

### Data analysis

Statistical analyses were performed using SPSS 26.0. Normally distributed measurement data were presented as mean ± standard deviation and analyzed using one-way ANOVA. Categorical data were presented as frequency counts and percentages (*n*, %) and were analyzed using the chi-square test. Multifactorial analysis was conducted using ordered logistic regression to identify independent influencing factors among significant single factors. Results were considered statistically significant at *p* < 0.05.

Growth mixture modeling (GMM) was conducted using Mplus 7.0. GMM is useful as it provides information about the growth factors (intercept and slope) of each trajectory. The growth factors are interpreted as usual in longitudinal modeling: the level of the outcome variable when time is equal to zero (intercept) and the rate of change in the outcome over time (slope). GMM operates under several key assumptions, including that within-class growth follows a specified parametric form (here, linear), that class membership is invariant over time, and that missing data are missing at random (MAR). It is important to acknowledge the potential limitations associated with these assumptions. First, the assumption of linear growth within each class may oversimplify the true developmental processes; non-linear trajectories (e.g., quadratic or piecewise growth) could exist but were not modeled, potentially biasing class enumeration and parameter estimates. Second, the assumption of time-invariant class membership implies that individuals do not transition between latent classes over the study period, which may not hold in dynamic developmental processes. Third, although MAR is a standard and relatively flexible assumption in longitudinal models, violations (e.g., missingness dependent on unobserved outcomes) could lead to biased estimates if not properly handled.

Model fit was assessed using the Akaike information criterion (AIC), Bayesian information criterion (BIC), and adjusted Bayesian information criterion (aBIC), with smaller values indicating better fit. Information entropy (ranging from 0 to 1) and the bootstrap likelihood ratio test (BLRT) were used to evaluate classification accuracy, with higher entropy values suggesting more accurate classification and a significant BLRT indicating that a k-class model fits significantly better than a k-1-class model. Model selection was further guided by theoretical interpretability, class sizes (avoiding classes comprising <5% of the sample), and posterior probabilities. We recognize that model selection relying on multiple fit indices can yield ambiguous solutions, as different indices may point to different class solutions—for instance, the BIC tends to favor more parsimonious models, whereas the BLRT may favor more complex ones.

### Sensitivity analysis

First, the alternative model setup was adopted for verification. To test whether the results were dependent on the assumption of “equal variances,” we conducted a latent profile analysis for the 1–4 categories using the variance-free model (allowing for free estimation of variances for each category). The results showed that the model fitting indicators still supported the 3-category model as the optimal one, and the trajectory patterns of the three categories (the reduction group, the fluctuation group, and the improvement group) were highly consistent with the results of the original analysis.

Second, cross-validation was conducted using the random sample splitting method. The total sample (*N* = 278) was randomly divided into two sub-samples (each containing 139 cases), and the latent profile analysis was independently performed on each sub-sample. The results showed that both sub-samples independently supported the 3-category model as the optimal model, and the category proportions and trajectory patterns were highly consistent with the results of the total sample.

Thirdly, Bootstrap resampling was used to test the stability of parameters. 5,000 Bootstrap resamplings were conducted on the three-class model to obtain the confidence intervals of the mean values for each category. The results showed that the range of the confidence intervals was reasonable, and there was no significant overlap among the categories, confirming the discrimination and stability of the three trajectories.

## Results

### Inclusion of subjects and data collection

**Figure 1:**
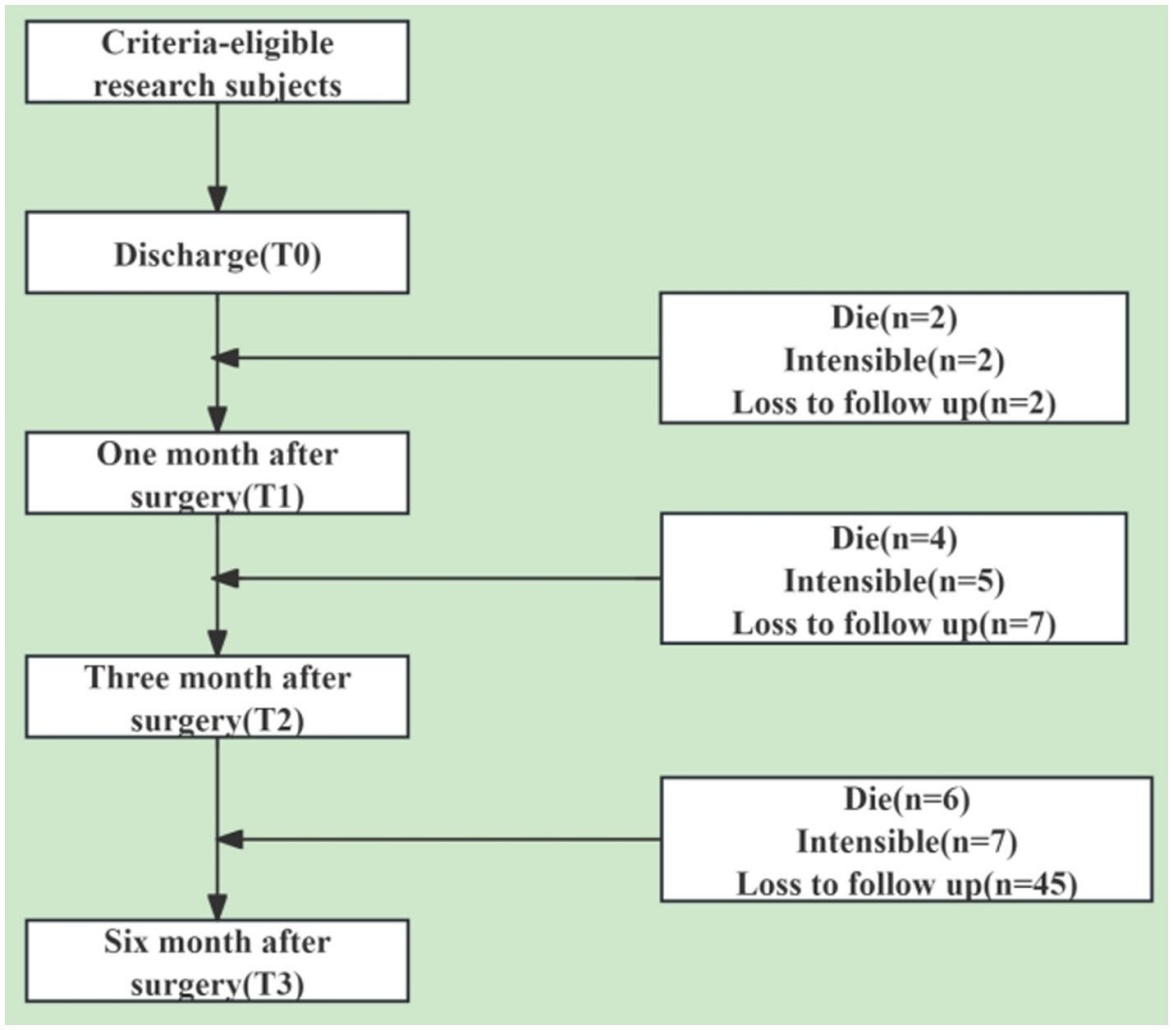
Sample attrition diagram.

A total of 358 study participants who met the criteria were included in this study, and the follow-up ended at 6 months postoperatively, with four time points, and the sample attrition is detailed in Figure 1.

Demographic information for the study sample is presented in Table 1. A total of 278 study subjects were finally included in this study. More than half participants (*n* = 157, 56.5%) had received at least a middle school education (over 9 years of education). In terms of marital status, most of the participants were married (*n* = 249, accounting for 89.6%), while only 7.9% (*n* = 22) were unmarried, and only 2.5% (*n* = 7) were divorced or widowed, suggesting that the marital structure was relatively stable. Among the types of disease, type A entrapment accounted for 47.1% (*n* = 131) and type B accounted for 52.9% (*n* = 147). A history of hypertension was present in 57.9% (*n* = 161) of patients with a history of hypertension and 42.1% (*n* = 117) of those without a history of hypertension.

**Table 1:** Demographic characteristics of participants (*N* = 278).

Variable	Categories	Frequency (*N*)	Percentage (%)
Gender	Male	221	79.5
	Female	57	20.5
Residence	City	255	91.7
	Countryside	23	8.3
Education	Elementary	80	28.8
	Middle school	157	56.5
	High school	29	10.4
	College	12	4.3
Status of employment	Employ	144	51.8
	Retired	111	39.9
	Other	23	8.3
Occupation	Business/enterprise	104	37.4
	Farmer	38	13.7
	Individual	25	9.0
	Unemployed/other	111	39.9
Marital status	Unmarried	22	7.9
	Married	249	89.6
	Divorced/widowed	7	2.5
Per capita household income	≤1,000	34	12.2
	1,000–2,000	13	4.7
	2,000–3,000	94	33.8
	≥3,000	137	49.3
Type of medical insurance	Self-payment/other	27	9.7
	Urban medical insurance	98	35.3
	Employee medical insurance	153	55.0
Type of AD	Type A	131	47.1
	Type B	147	52.9
History of hypertension	Yes	161	57.9
	No	117	42.1
History of diabetes	Yes	1	0.4
	No	277	99.6

### Potential category analysis of health promoting behavior in patients after AD surgery

In the initial GMM model, there was significant variance in the intercept and slope (*p* < 0.001), indicating significant variability in both the mean health promoting behavior and the linear growth trajectory over 6 months. Based on the initial model, the number of classes in the model gradually increases until the best model fits the data. The results showed that the AIC, BIC, and aBIC indicators were higher in the 4-classes model than in the other models. The entropy of the 4-classes model was more than 0.8, and the LMR reached have no significant levels (*p* > 0.05), so it was not considered. The entropy value of the 3-classes model was smaller than that of the 2-classes model. The AIC, BIC, and aBIC values were smaller in the 3-classes model than in the 2-classes model. Moreover, when three classes were retained, LMR and BLRT reached significant levels (*p* < 0.001), entropy values were more desirable (>0.8), and the class probability was reasonable (all > 5%). The fitting index results are shown in Table 2.

**Table 2:** Summary of model fit statistics of GMM with different classes (*N* = 278).

Model	AIC	BIC	aBIC	Entropy	*p*		Category probability (%)			
					LMR	BLRT	1	2	3	4
1	8,984.753	9,013.774	8,988.407							
2	8,528.158	8,575.318	8,534.096	0.972	0.000	0.000	0.342	0.658		
3	8,158.409	8,223.707	8,166.631	0.985	0.000	0.000	0.338	0.432	0.230	
4	7,792.673	7,876.109	7,803.178	0.977	0.316	0.000	0.183	0.230	0.183	0.403

**Figure 2:**
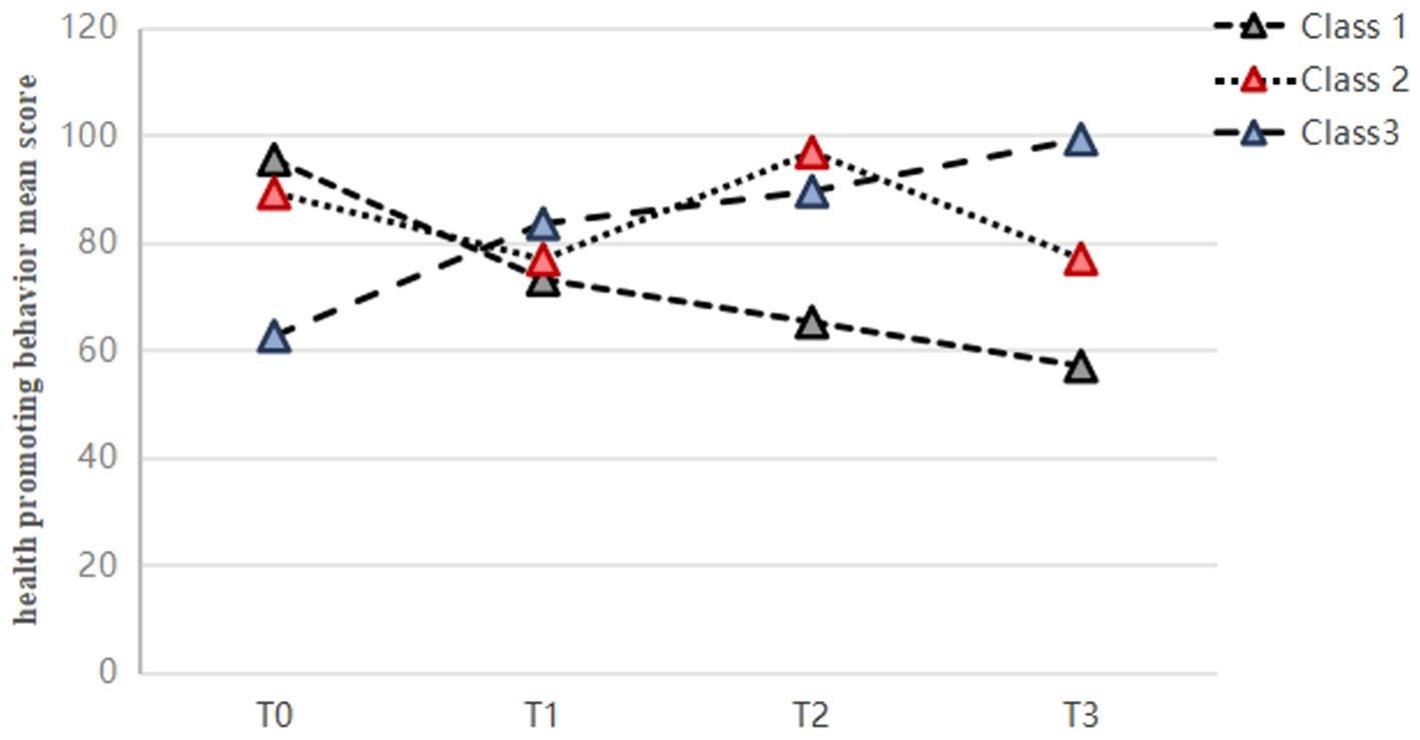
Three classes trajectory model for health promoting behavior from baseline to 6 months (*n* = 278). Discharge (T0), 1 month (T1), 3 months (T2), and 6 months (T3).

The three identified trajectories displayed distinct clinical patterns: Class 1 (33.8%), characterized by low baseline levels with significant negative slope, represented patients with persistently poor behavioral adherence requiring intensive intervention; Class 2 (43.2%), showing medium baseline with fluctuating patterns, corresponded to patients with inconsistent engagement typical of transitional recovery phases; Class 3 (23.0%), exhibiting low initial levels but significant positive growth, captured patients demonstrating substantial behavioral improvement through rehabilitation. These patterns were validated through consultation with cardiac rehabilitation specialists, who confirmed alignment with observed clinical phenotypes in post-discharge care. Combined with mean posterior classification probabilities of 0.87 and distinct baseline characteristic distributions across classes, the 3-class solution was determined to be both statistically robust and clinically actionable for tailoring differentiated nursing strategies. Three trajectories are shown in Figure 2.

### Univariate analysis of health promoting behavior in AD patients

The univariate analysis explored the factors affecting health promoting behavior trajectories. The results showed significant differences in the distribution of patients on age, education, Residence and History of hypertension for the three trajectories (*p* < 0.05). The results are detailed in Table 3.

**Table 3:** Demographic and characteristics by latent class (*N* = 278).

Variable	Class 1	Class 2	Class 3	*F*/*χ*^2^	*p*
Gender				2.902	0.234
Male	80 (85.1%)	93 (77.5%)	48 (75.0%)		
Female	14 (14.9%)	27 (22.5%)	16 (25.0%)		
Age	63.53 ± 10.872	56.87 ± 11.108	53.06 ± 12.195	17.947	*p* < 0.001
Residence				8.605	**0.014**
City	80 (85.1%)	113 (94.2%)	62 (96.9%)		
Countryside	14 (14.9%)	7 (5.8%)	2 (3.1%)		
Education				29.469	***p* < 0.001**
Elementary	36 (38.3%)	38 (31.7%)	6 (9.4%)		
Middle school	53 (56.4%)	65 (54.2%)	39 (60.9%)		
High school	2 (2.1%)	12 (10.0%)	15 (23.4%)		
College	3 (3.2%)	5 (4.2%)	4 (6.3%)		
Status of employment				7.422	0.115
Employ	42 (44.7%)	62 (51.7%)	40 (62.5%)		
Retired	45 (47.9%)	49 (40.8%)	17 (26.6%)		
Other	7 (7.4%)	9 (7.5%)	7 (10.9%)		
Occupation				9.829	0.132
Business/enterprise	29 (30.9%)	48 (40.0%)	27 (42.2%)		
Farmer	13 (13.8%)	11 (9.2%)	14 (21.9%)		
Individual	9 (9.6%)	12 (10.0%)	4 (6.3%)		
Unemployed/other	43 (45.7%)	49 (40.8%)	19 (29.7%)		
Marital status				0.667	0.955
Unmarried	7 (7.4%)	10 (8.3%)	5 (7.8%)		
Married	84 (89.4%)	108 (90.0%)	57 (89.1%)		
Divorced/widowed	3 (3.2%)	2 (1.7%)	2 (3.1%)		
Per capita household income				3.563	0.736
≤1,000	10 (10.6%)	16 (13.3%)	8 (12.5%)		
1,000–2,000	6 (6.4%)	6 (5.0%)	1 (1.6%)		
2,000–3,000	34 (36.2%)	41 (34.2%)	19 (29.7%)		
≥3,000	44 (46.8%)	57 (47.5%)	36 (56.3%)		
Type of medical insurance				6.047	0.196
Self-payment/other	13 (13.8%)	9 (7.5%)	5 (7.8%)		
Urban medical insurance	25 (26.6%)	48 (40.0%)	25 (39.1%)		
Employee medical insurance	56 (59.6%)	63 (52.5%)	34 (53.1%)		
Type of AD				3.232	0.199
Type A	38 (40.4%)	58 (48.3%)	35 (54.7%)		
Type B	56 (59.6%)	62 (51.7%)	29 (45.3%)		
History of hypertension				28.137	***p* < 0.001**
Yes	69 (73.4%)	72 (60.0%)	20 (31.3%)		
No	25 (26.6%)	48 (40.0%)	44 (68.8%)		
History of diabetes				1.965	0.374
Yes	1 (1.1%)	0 (0.0%)	0 (0.0%)		
No	93 (98.9%)	120 (100.0%)	64 (100.0%)		

### Logistic regression analysis of health promoting behavior in AD patients

Table 4 shows the comparison between the medium-volatility group and the low-stability group. The results of Logistic regression analysis show that age (OR = 0.950, 95%CI: 0.925–0.975, *p* < 0.001) and whether there is a history of hypertension (OR = 1.941, 95%CI: 1.049–3.590, *p* = 0.035) were significant influencing factors in the medium-volatility group. The comparative Logistic regression analysis results between the high-increasing group and the low-stability group showed that age (OR = 0.923, 95%CI: 0.892–0.954, *p* < 0.001), educational level (OR = 2.507, 95%CI: 1.503–4.182, *p* < 0.001) and a history of hypertension (OR = 6.613, 95%CI: 3.056–14.313, *p* < 0.001) were both significant influencing factors.

**Table 4:** Multinomial logistic regression results of different trajectories (*N* = 278).

	Variables	*B*	Std. Error	Wald	df	*p*-value	OR	95% OR	
								Lower bound	Upper bound
Class 2	Constant	2.764	1.148	5.799	1	0.016			
	Age	−0.051	0.013	14.558	1	*p* < 0.001	0.950	0.925	0.975
	Residence	−0.766	0.506	2.288	1	0.130	0.465	0.172	1.254
	Education	0.293	0.224	1.713	1	0.191	1.340	0.865	2.076
	History of hypertension	0.663	0.314	4.462	1	0.035	1.941	1.049	3.590
Class 3	Constant	0.891	1.526	0.341	1	0.559			
	Age	−0.081	0.017	21.681	1	*p* < 0.001	0.923	0.892	0.954
	Residence	−1.072	0.852	1.583	1	0.208	0.342	0.064	1.819
	Education	0.919	0.261	12.395	1	*p* < 0.001	2.507	1.503	4.182
	History of hypertension	1.889	0.394	22.999	1	*p* < 0.001	6.613	3.056	14.313

Compare to low-stability group; Class 2 = medium-volatility group; Class 3 = high-increasing group; *P* < 0.05 indicates statistical significance; *P* > 0.05 indicates no statistical significance.

## Discussion

In this study, we utilized the health-promoting behavior scale to track changes in the health-promoting behavior levels of AD patients within 6 months post-surgery. GMM was used to analyze the trajectories of health-promoting behavior changes in patients at different time points after surgery. The results of the GMM showed population heterogeneity in health-promoting behavior changes after AD surgery. Upon completion of the model fitting process, three discrete behavioral trajectory clusters were delineated, specifically characterized as the “low-stability group,” the “medium-volatility group,” and the “high-increasing group.” Statistical analysis revealed significant intergroup disparities among these cohorts. Furthermore, the sociodemographic and clinical data of AD patients were comprehensively collected to explore factors related to the trajectory variation among different subgroups of AD patients.

This study identified three distinct trajectories of health-promoting behavior following aortic coarctation surgery: low-stable (33.8%), moderate-fluctuating (43.2%), and high-increasing (23.0%). These patterns align with Yu et al.’s findings in pregnant women (17) and carry clinical relevance for postoperative risk stratification. The low-stable group, scoring below 40% on behavioral indices, mirrors the “high cardiovascular risk profile” identified by Morris et al. (18, 19) suggesting vulnerability to poor blood pressure control and recurrent interventions. Conversely, the high-increasing group approaches the >75% threshold of “optimal cardiovascular health” (20), indicating favorable prognosis. From a theoretical standpoint, these trajectories map onto Prochaska’s stages of change: precontemplation/contemplation stagnation (low-stable), action-maintenance oscillation (moderate-fluctuating), and successful maintenance (high-increasing). Unmeasured disease perception, psychological distress, and social support likely drive these differential patterns (18, 21). Though their predictive effects require empirical validation. Future research should link trajectory membership to hard clinical endpoints—reintervention rates, blood pressure targets, and major adverse cardiovascular events—to establish prognostic utility. Additionally, stepped-care interventions tailored to trajectory risk profiles (intensive motivational interviewing for low-stable, relapse prevention for moderate-fluctuating, maintenance support for high-increasing) may translate these statistical classifications into actionable clinical tools.

This study found that age was influence factors in the medium-volatility and high-increasing group compared to the low-stability group, consistent with similar research (22). The causes of this phenomenon can be attributed to the following two aspects. First, with advancing age, there is a gradual decline in physical function, and the structure and function of the aortic wall also undergo changes (23). This reduces the body’s adaptability and tolerance to health-promoting behaviors in older adult(s) patients with aortic dissection, thereby decreasing their ability and willingness to engage in such behaviors (13). Second, corroborating studies (24) have indicated that the scores for physical function, role function, and other dimensions in aortic dissection patients tend to decline with increasing age. This further confirms that as patients grow older, their overall health status and functional state deteriorate, making it difficult for them to actively practice health-promoting behaviors.

The results show that AD patients with hypertension are 1.95 times more likely to be in the moderate-volatility group and 6.613 times more likely to be in the high-increase group than those without hypertension. The reasons for this phenomenon may be as follows. First, from a psychological perspective, it has been suggested that patients with a history of hypertension may have a stronger awareness of their health risks due to their long-term struggle with the disease (25). This heightened risk awareness, potentially mediated by disease perception and illness coherence, was not directly assessed in our study but represents an important psychological mechanism warranting future investigation. For example, based on previous research, such patients may realize the importance of health-promoting behaviors in preventing disease progression and complications, and thus are more likely to actively adopt and maintain such behaviors. Patients may realize the importance of health-promoting behaviors in preventing disease progression and complications, and thus are more likely to actively adopt and maintain such behaviors. Second, in terms of disease management, patients with hypertension usually have more frequent interactions with the healthcare system, such as regular check-ups and medication adjustments (26). During these interactions, patients may receive more information and guidance on health-promoting behaviors, which can increase their likelihood of adopting positive health behaviors (27). The quality and continuity of these interactions, potentially influenced by patient-provider communication and trust, constitute contextual factors that merit inclusion in subsequent studies. Third, from a social support perspective, it has been proposed that patients with hypertension may have more support from family and friends in managing their health, which can also encourage them to adopt health-promoting behaviors (28). This informal support network, distinct from formal healthcare resources, represents a critical contextual variable that should be operationalized and tested in future longitudinal research.

The results of this study found that patients with high levels of education were more likely to be in the high-increasing group compared to the low-stability group. The present study found that among patients with aortic constriction, those with higher levels of education were more likely to belong to the “high-increasing group” than to the “low-stability group.” This finding may reflect the profound impact of educational background on disease management ability. Higher education is generally associated with greater health literacy (29), enabling patients to better understand complex medical information, follow individualized treatment recommendations (e.g., strict blood pressure monitoring, medication adherence, or lifestyle interventions), and participate more proactively in clinical decision-making (30). In addition, a high level of education may be indirectly associated with better socioeconomic status, which improves access to medical resources (e.g., regular specialty follow-up, cardiac rehabilitation programs) and psychosocial resources to cope with the stress of the disease (31, 32). These psychosocial resources, including coping strategies, resilience, and perceived control, represent important psychological mediators that were not assessed in our study but may explain why educational advantage translates into superior behavioral trajectories. It is important to note that while these factors, such as socioeconomic status and psychosocial resources, were not directly assessed in the current study, they have been identified in previous research as important contributors to disease management and health outcomes (31, 32). This combined ability may have facilitated more active disease management behaviors, which ultimately manifested in a more significant trajectory of clinical improvement (the “high-growth group”). The absence of these psychological and contextual variables in our analytical model constitutes a significant limitation, as we cannot rule out confounding or mediating effects of unmeasured factors on the observed associations between age, hypertension, education, and trajectory membership. For example, the protective effect of education may be partially attributable to unmeasured correlates such as cognitive function, self-efficacy, or social capital, rather than education per se. The results emphasize the need to assess education level and socioeconomic factors in managing aortic constriction long-term. They indicate that developing supportive, simplified health education and better follow-up systems for less educated patients can improve outcomes for all.

### Limitations

However, this study has several limitations. First, patients were recruited using convenience sampling from the cardiac surgery ward of only one tertiary hospital in China, which may restrict the generalizability of our findings to other settings or populations. Second, data were collected through self-report, and therefore, the possibility of response bias could not be eliminated. Third, the relatively small sample size may have resulted in selection bias and reduced statistical power. Fourth, as an observational study, no causal inferences could be determined. Additionally, we did not assess important psychological and social variables, such as illness perception, anxiety, depression, or social support, which may represent significant confounders. Future studies should incorporate these psychobehavioral factors to strengthen explanatory depth, reduce residual confounding, and provide a more comprehensive understanding of the phenomena under investigation.

## Conclusion

This study identified three potential categories, divided into low-stability medium-volatility and high-increasing group, indicating differences in health-promoting behavior change in patients after AD surgery. Age, educational attainment, and history of hypertension are significant factors influencing patients’ health promotion behaviors. The findings of this study fill a current research gap and provide valuable insights for optimizing patient health behavior management in AD surgery. Future studies should incorporate additional follow-up time points and extended follow-up periods to comprehensively assess patients’ long-term health-promoting behavioral changes, thereby providing more guidance for clinical practice.

## Data Availability

The raw data supporting the conclusions of this article will be made available by the authors, upon reasonable request to the corresponding author at: 15960815442@163.com.
